# Resource-Constrained Specific Emitter Identification Based on Efficient Design and Network Compression

**DOI:** 10.3390/s25072293

**Published:** 2025-04-04

**Authors:** Mengtao Wang, Shengliang Fang, Youchen Fan, Shunhu Hou

**Affiliations:** 1 Graduate School, Space Engineering University, Beijing 101416, China; 13373422473@163.com (M.W.); hshhsc2022@163.com (S.H.); 2 School of Space Information, Space Engineering University, Beijing 101416, China

**Keywords:** specific emitter identification (SEI), radio frequency fingerprinting (RFF), deep learning (DL), lightweight convolution network (LCNet), sparse feature selection (SFS)

## Abstract

Specific emitter identification (SEI) methods based on deep learning (DL) have effectively addressed complex, multi-dimensional signal recognition tasks by leveraging deep neural networks. However, this advancement introduces challenges such as model parameter redundancy and high feature dimensionality, which pose limitations for resource-constrained (RC) edge devices, especially in Internet of Things (IoT) applications. To tackle these problems, we propose an RC-SEI method based on efficient design and model compression. Specifically, for efficient design, we have developed a lightweight convolution network (LCNet) that aims to balance performance and complexity. Regarding model compression, we introduce sparse regularization techniques in the fully connected (FC) layer, achieving over 99% feature dimensionality reduction. Furthermore, we have comprehensively evaluated the proposed method on public automatic-dependent surveillance-broadcast (ADS-B) and Wi-Fi datasets. Simulation results demonstrate that our proposed method exhibits superior performance in terms of both recognition accuracy and model complexity. Specifically, LCNet achieved accuracies of 99.40% and 99.90% on the ADS-B and Wi-Fi datasets, respectively, with only 33,510 and 33,544 parameters. These results highlight the feasibility and potential of our proposed RC-SEI method for RC scenarios.

## 1. Introduction

### 1.1. Background

Sensors, as a crucial component of the IoT ecosystem, play a pivotal role in converting data from the physical world into actionable information for network systems. This data conversion has fueled the intelligent development of various industries [1]. In the transportation sector, sensors are employed for intelligent traffic control. In agriculture, they are utilized for monitoring soil moisture and crop health. In healthcare, sensors enable real-time monitoring of patients’ vital signs. With ongoing technological advancements, sensors are progressively evolving towards miniaturization, low power consumption, and enhanced integration [2]. However, due to the inherent resource constraints of sensor nodes, simplified authentication mechanisms are often employed [3]. For instance, at the network and data link layers, device identification typically relies on Internet Protocol (IP) addresses or Media Access Control (MAC) address identifiers. However, IP addresses are not unique, and MAC addresses are susceptible to tampering. This vulnerability presents substantial security challenges [4] for sensor identity recognition, including but not limited to impersonation attacks, Sybil attacks, and risks of privacy breaches.

**Figure 1:**
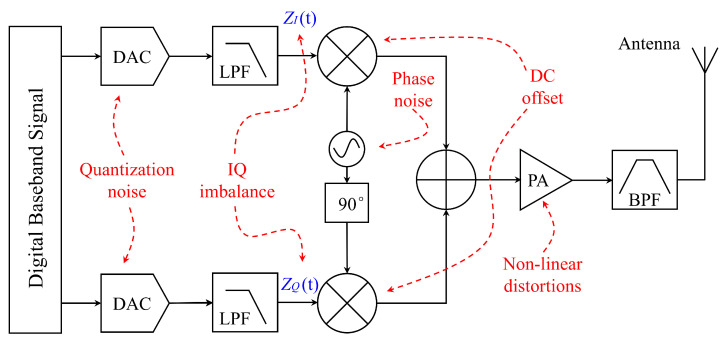
RFF generation mechanism (adapted from [10]).

To address these security vulnerabilities, SEI technology offers a viable solution. SEI is a physical layer-based technique [5] that identifies and differentiates between various wireless transmitters, thereby effectively preventing unauthorized access and interference [6]. Specifically, SEI typically utilizes radio frequency fingerprinting (RFF) for implementation. As illustrated in Figure 1, RFF represents an inherent physical characteristic of a device, stemming from intrinsic imperfections in the transmitter hardware, such as the non-linearity of the power amplifier [7]. These fingerprints are inherently difficult to replicate or forge. By extracting these unique features (i.e., fingerprints) of the device, SEI provides a robust method of authentication, thereby ensuring the authenticity and security of device identity [8]. This approach offers the advantage of low computational overhead and minimal memory capacity requirements in RC devices [9].

Based on the collected signal used to identify emitters, SEI methods can be categorized into two main types [11]: (1) SEI methods based on transient signals, which utilize signals during the transition period from the transmitter turning on to full amplitude (or the reverse process). During this transition period, the transmitter generates time-varying effects and instantaneous distortions, resulting in characteristics such as carrier frequency offset (CFO) and phase deviation. However, this method necessitates a high sampling rate and precise starting point detection [12], rendering it less suitable for RC-IoT scenarios. (2) SEI methods using steady-state signals, which are more readily captured than transient signals. This second type represents the primary research direction at present and is also the focus of this paper.

### 1.2. Motivations

SEI methods employing steady-state signals generally consist of three stages: pre-processing, feature extraction, and recognition. The pre-processing stage typically encompasses operations such as filtering, synchronization, denoising, and power normalization. Feature extraction and recognition constitute the key stages in SEI. Tu et al. [13] proposed an RFF algorithm utilizing four statistical features across the time, wavelet, Gabor transform, and Wigner–Ville distribution domains. Subsequently, the features were then reduced in dimensionality via Robust Principal Component Analysis (RPCA) and classified using a Support Vector Machine (SVM). While this method has proven effective for specific IoT devices, it relies on manually designed features, necessitating substantial domain expertise. Chen et al. [14] introduced an RFF identification method using the Short-Time Fourier Transform (STFT) to extract energy and periodic characteristics from transient signals of wireless modules, also employing SVM for classification. However, its reliance on specific transceiver module transient signal characteristics limits its generalizability.

In recent years, DL has demonstrated robust data analysis capabilities across various domains [15], including diverse research directions within SEI. In the common scenario of SEI, Yu et al. [16] applied a multi-resolution convolution neural network (MSCNN) to identify 54 CC 2530 devices, achieving an identification accuracy exceeding 97% under high signal-to-noise ratio (SNR) conditions. In the dynamic scenario of SEI, Han et al. [17] proposed a two-stage cross-domain identity recognition model and introduced a triple transfer strategy, effectively enabling rapid model reconstruction under dynamic conditions. In few-shot (FS) SEI, Wang et al. [18] developed a method based on deep metric ensemble learning (DMEL), achieving an identification accuracy exceeding 98% with more than five samples per class for ADS-B signals. In semi-supervised SEI, Xue et al. [19] introduced a semi-supervised SEI method using metric adversarial training (MAT), achieving an identification accuracy of 84.8% when labeled samples constituted 10% of all samples in ADS-B signals. In open-set SEI, Huang et al. [20] utilized the metric-enhanced denoising autoencoder (MEDAE) architecture to propose a robust SEI method, demonstrating noise resilience and exceptional accuracy in ADS-B data.

Indeed, the recent surge in enthusiasm among researchers for DL is largely attributed to its provision of multi-layer computational models. These models have enabled a powerful data-driven approach to address many problems traditionally considered challenging. However, to achieve enhanced performance and functionality, these models are becoming increasingly complex. The substantial memory and computational requirements hinder their deployment in RC-IoT scenarios. Consequently, we propose a versatile lightweight RC-SEI method.

### 1.3. Related Works

To address the aforementioned challenges, numerous approaches have been proposed by researchers in recent years. Based on the methodologies outlined by Guo et al. [21] for mitigating DL complexity problems, these approaches can be classified into two main categories: (1) Efficient Design, which focuses on minimizing redundancy in neural networks from the outset of the design process. Examples of such techniques include replacing FC layers with global average pooling (GAP) layers [22] and substituting standard convolution layers with group convolution layers [23]. (2) Network compression, which aims to prune or compress the model weights during or after training through techniques such as quantization [24], network pruning [25], and low-rank approximation [26], thereby reducing storage space and computational costs. Subsequently, the work associated with these two categories of methods will be described in detail.

(1) Efficient design. Zhang et al. [27] proposed an SEI method based on a single-layer feedforward network (SFEBLN), which can efficiently identify radiation sources on a CPU, achieving an exponential improvement in computational efficiency and eliminating dependence on GPUs. Chan et al. [23] introduced a novel lightweight single-stream neural network composed of group convolution layers and transformer encoding, achieving a 16-fold scale reduction with only a 0.12% decrease in accuracy. Dong et al. [28] presented a lightweight distributed learning approach based on a multichannel multifunctional spatiotemporal hybrid deep neural network (MCMBNN), which can be effectively deployed in edge devices. Hua et al. [29] developed an adaptive feature composition method based on knowledge graphs and attention mechanisms, significantly reducing model complexity and achieving an average recognition accuracy above 99.2% at the optimal compression ratio.

(2) Network compression. Dong et al. [24] proposed an edge-learning-based collaborative automatic modulation classification (C-AMC) framework. This framework employs post-training quantization, mapping weights to b-bit integers to reduce model size and accelerate inference. This approach enables significant model space compression with a slight sacrifice in accuracy. Tao et al. [30] introduced a lightweight SEI method based on sparse regularization, which prunes FC layers during model training to create a more compact network. Jiang et al. [25] introduced a complex-valued soft-log threshold reweighting (CV-SLTR) algorithm. This algorithm significantly reduces the number of model parameters and computational complexity by designing complex-valued soft threshold and log-sum threshold operators for weight pruning. Li et al. [31] presented a multi-constrained model compression (MCMC) method. Leveraging reinforcement learning, the method automatically searches for the pruning rate for each layer, enabling channel pruning of the neural network. Applied to the VGG-16 network, this method achieved an 80% reduction in FLOPs, a 2.31× reduction in memory usage, and a 1.92× acceleration.

In addressing the complexity of DL, Guo et al. [21] contend that both efficient design and network compression are indispensable steps prior to the implementation and deployment of networks. However, the majority of lightweight SEI research based on DL has focused on either one of these approaches, which still presents significant room for optimization. Consequently, this paper proposes an efficient lightweight RC-SEI method, incorporating both efficient design and network compression principles.

### 1.4. Main Contributions

In this paper, we propose an efficient and lightweight RC-SEI approach to address the IoT authentication security challenges. The main contributions are summarized as follows:

- We propose a lightweight convolution network, LCNet. The implementation of strategies such as complex convolution, depth-wise separable convolution, and attention mechanisms can enhance model performance while reducing complexity.
- We introduce a sparse feature selection (SFS) framework for RC-SEI. Specifically, the incorporation of scaling factors and corresponding sparse regularization in the FC layer enable effective model compression.
- We evaluated the proposed method using the open source real-world ADS-B dataset [32] and the Wi-Fi [33] dataset. Furthermore, we compared it against several state-of-the-art networks, including ULCNN [22], CVNN [24], MCNet [34], and MCLDNN [35].
- Based on the effectiveness of the RC-SEI method proposed in this paper, we suggest a standardized procedure for designing lightweight network models.

## 2. System Model and Problem Formulation

### 2.1. System Model

**Figure 2:**
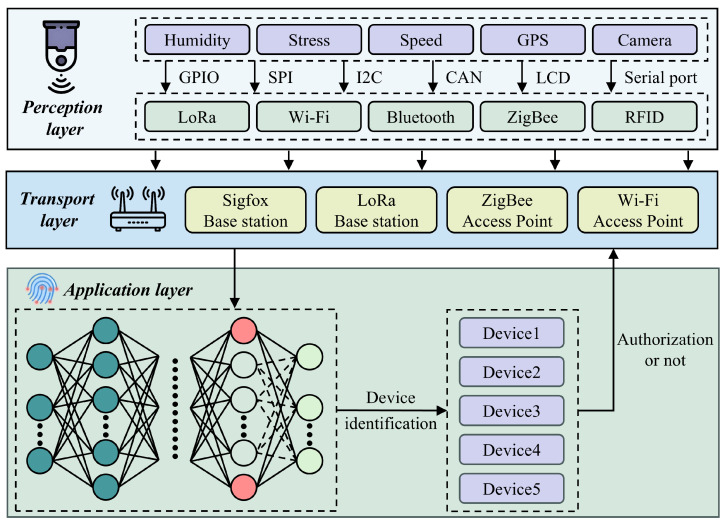
SEI-based secure identification system for IoT devices.

As illustrated in Figure 2, the SEI-based IoT device identity identification system model comprises the following layers: perception layer, transport layer, and application layer. The perception layer is composed of sensors, wireless terminals, and other devices that are primarily responsible for data collection and processing. The transport layer consists of routers, base stations, access points, and other devices, which are primarily responsible for the transmission and forwarding of data. The application layer employs the RC-SEI method to achieve secure identification of device identities. In this study, the focus is on the application layer.

### 2.2. Problem Formulation

#### 2.2.1. SEI Problem

Based on the aforementioned system model and the signal dataset, the received radio frequency (RF) signal in this article can be expressed as follows [18]:

(1) $$x_{i}\left(t\right)=s_{i}\left(t\right)*h_{i}\left(t\right)+n_{i}\left(t\right),\;i=1,2,\ldots ,N,$$

where $x_{i}\left(t\right)$ represents the received RF signal, $s_{i}\left(t\right)$ represents the RF signal input to the channel after modulation, $h_{i}\left(t\right)$ represents the channel impulse response between the transmitter and receiver, $n_{i}\left(t\right)$ represents the additive white Gaussian noise, and ∗ represents the convolution operation. This article assumes that the dataset is defined as ${\left\{x_{i},y_{i}\right\}}_{i=1}^{N}$, where $y_{i}$ represents the label corresponding to different emitters. Therefore, the DL-based SEI method can be defined as a multi-classification recognition problem based on the maximum a posteriori (MAP) criterion, which can be expressed as follows:

(2) $$\hat{y}=\underset{y\in Y}{\arg\max}f\left(y|x;w\right),$$

where *y* and $\hat{y}$ represent the true and predicted categories, respectively. f(·) is the mapping function between input samples and labels, and w represents the weights of the network. In this process, DL constructs the mapping function f(·) based on a substantial number of labeled samples. Through multiple training iterations, it minimizes the error between the predicted category $\hat{y}$ and the true category *y* under the supervision of the cross-entropy loss function $L_{CE}$. This is done to obtain the optimal network weights w. Consequently, the objective function of the classification problem can be expressed as follows:

(3) $$\min L_{CE}\left(\hat{y},y\right).$$

#### 2.2.2. RC-SEI Problem

In the RC-SEI problem, efficient model design is essential, and model compression is equally crucial. FC layers are the most prevalent neural network layers in models, capable of learning all input data combinations. However, FC layers have a large number of parameters, leading to high computational costs and potential overfitting. Therefore, our objective is to maximize the feature sparsity of the FC layer, which enables effective network compression.

Specifically, sparse parameters $w_{SF}$ are introduced into the FC layer, and the corresponding weights are pruned when the components of $w_{SF}$ are equal to zero. Furthermore, sparse regularization term R(·) is incorporated into the loss function to restrict more components of $w_{SF}$ to zero. Thus, the objective function can be expressed as follows [30]:

(4) $$\min\left[L_{CE}\left(\hat{y},y\right)+R\left(w_{SF}\right)\right].$$

## 3. The Proposed RC-SEI Method

In this section, we provide a detailed description of the proposed RC-SEI method. The approach comprises three principal components: (1) An efficient design of lightweight convolution structure; (2) Selection of sparse features for network compression; (3) Accelerating loss convergence with accelerate proximal gradient detachment (APGD), which is based on the Nesterov accelerated gradient (NAG) algorithm.

### 3.1. Lightweight Convolution Network Architecture

Drawing inspiration from work in [22] and our previous study [36], we designed an efficient, lightweight convolution network. As detailed in Table 1, the network architecture consists of two main components: (1) An in-phase and quadrature channel fusion (IQCF) module based on complex-valued (CV) convolution; (2) A lightweight convolution module (LCM) based on depth-wise separable convolution and channel attention. The IQCF module effectively extracts coupled features from IQ signals, while the LCM extracts deep sample features and reduces feature dimensions.

#### 3.1.1. IQCF

In this study, the sample x consists of IQ signals. Due to the displacement effect, the real and imaginary components of the IQ signal interact under any phase variation [37]. However, conventional real-valued (RV) convolution models treat the real and imaginary parts of IQ signals as independent, ignoring their inherent coupling characteristics. Therefore, IQCF employs CV operations instead of RV ones. This approach not only maximally preserves the original data information but also reduces the number of parameters used. The formula for CV convolution can be expressed as follows [25]:

(5) $$\begin{array}{cc} W_{\mathrm{CV}}*x & =R\left(W_{\mathrm{CV}}\right)*R\left(x\right)-I\left(W_{\mathrm{CV}}\right)*I\left(x\right)+j\cdot \left[R\left(W_{\mathrm{CV}}\right)*I\left(x\right)+I\left(W_{\mathrm{CV}}\right)*R\left(x\right)\right], \end{array}$$

where $W_{\mathrm{CV}}$ represents the weights of CV convolution, and R(·) and I(·) represent the real and imaginary parts, respectively. Moreover, we calculate the number of parameters and floating point operations (FLOPs) of CV convolution. For comparison, we also introduce the RV convolution.

Given an input channel of 2 and a convolution kernel size of $K_{C}$, the shape of the output feature map of the convolution is $2M\times L_{out}$. It is important to note that, in CV convolution, the 2M channels are equally divided between the real and imaginary channels. In contrast, all channels in RV convolutions are RV. The parameters and FLOPs for CV convolution ($P_{CV}$, $T_{CV}$) and RV convolution ($P_{RV}$, $T_{RV}$) are given by the following formulas [38]:

(6) $$P_{CV}=2\times K_{C}\times M\times 2,$$

(7) $$T_{CV}=2\times K_{C}\times M\times L_{out}\times 4+2\times M\times L_{out},$$

(8) $$P_{RV}=K_{C}\times 2M\times 2,$$

(9) $$T_{RV}=2\times 2\times K_{C}\times 2M\times L_{out}.$$

As indicated by (6) to (9), $P_{CV}=P_{RV}$, $T_{CV}/T_{RV}=1+1/4M$. This implies that, while CV and RV convolutions have the same number of parameters, the CV convolution requires more FLOPs. Therefore, to minimize the overall FLOPs of the model, only one layer of the IQCF module is employed. Furthermore, it is crucial to note that bias parameters for the convolution kernels are omitted during model training. Consequently, the parameter count calculations presented above do not include bias parameters.

#### 3.1.2. LCM

**Figure 3:**
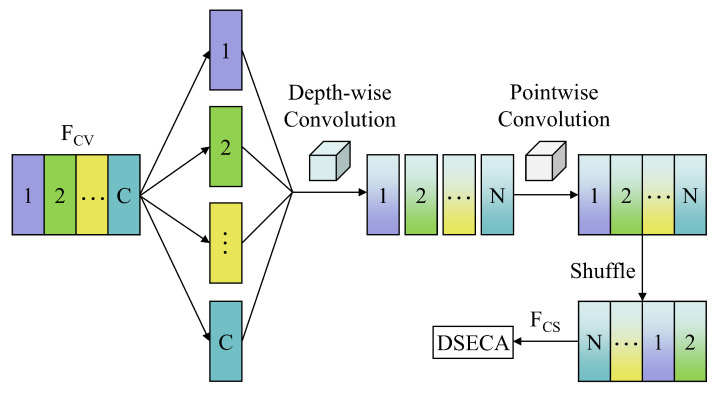
The structure of LCM.

As depicted in Figure 3, the LCM comprises three main components: depth-wise separable convolution, channel shuffle, and dual-stream efficient channel attention (DSECA). Depth-wise separable convolution is a common technique for lightweight design, notably in the MobileNet series [39]. This approach separates the mapping of cross-channel correlations and spatial correlations, leading to improved performance with reduced computations. Specifically, during the depth-wise convolution stage, the operation is applied independently to each input channel. In the pointwise convolution stage, a 1×1 convolution kernel integrates information across channels to produce the final output feature maps.

However, feature exchange between channels is challenging in the output feature maps. To address this, channel shuffle is introduced to facilitate feature transfer between different channels. This operation effectively disrupts dependencies between features, thereby helping the network to learn more robust and diverse feature representations. After channel shuffle, the output feature map $F_{CS}$ is obtained. Additionally, the DSECA module is applied to each LCM layer to enhance key feature extraction.

#### 3.1.3. DSECA

**Figure 4:**
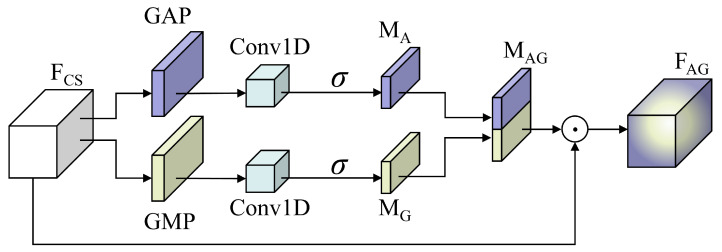
The structure of DSECA, where σ is the sigmoid activation function.

DSECA, an enhanced version of ECA [40], introduces an additional global max pooling (GMP) stream for a more comprehensive attention mechanism. As shown in Figure 4, DSECA utilizes global average pooling (GAP) and GMP to extract complementary features from $F_{CS}$. Specifically, GAP extracts aggregated features $M_{A}$, and GMP extracts salient features $M_{G}$. The comprehensive attention channel $M_{AG}$ is then formed by vertically concatenating $M_{A}$ and $M_{G}$. Eventually, the attention feature map $F_{AG}=F_{CS}⊙M_{AG}$ is obtained, where ⊙ represents the Hadamard product. This design effectively suppresses less important features. Furthermore, DSECA contains only eight learnable parameters, ensuring extremely low computational cost.

Since channel shuffle is parameter-free, we focus on analyzing the complexity of separable convolution in LCM. To facilitate a comparison, we introduce standard 1D convolution (Conv1D). Let the kernel size be $K_{C}$, the input and output channels be $C_{in}$ and $C_{out}$, respectively, and the output feature map size be $L_{out}$. In that case, the comparison between standard convolution and separable convolution is as follows [41]:

(10) $$\frac{P_{sep}}{P_{std}}=\frac{K_{C}\times C_{in}+C_{in}\times C_{out}}{K_{C}\times C_{in}\times C_{out}}=\frac{1}{C_{out}}+\frac{1}{K_{C}},$$

(11) $$\begin{array}{cc} \frac{T_{sep}}{T_{std}} & =\frac{K_{C}\times L_{out}\times C_{in}+L_{out}\times C_{in}\times C_{out}}{K_{C}\times L_{out}\times C_{in}\times C_{out}}=\frac{1}{C_{out}}+\frac{1}{K_{C}}. \end{array}$$

$P_{sep}/P_{std}$ and $T_{sep}/T_{std}$ represent the parameter and FLOPs ratios between separable and standard convolution, respectively. Within the LCM, where $K_{C}=5$, $C_{out}=64$. Consequently, we can calculate that $P_{sep}/P_{std}$ and $T_{sep}/T_{std}$ both are 0.216. This shows that separable convolution reduces complexity by nearly 80% compared to standard convolution.

### 3.2. Sparse Feature Selection

**Figure 5:**
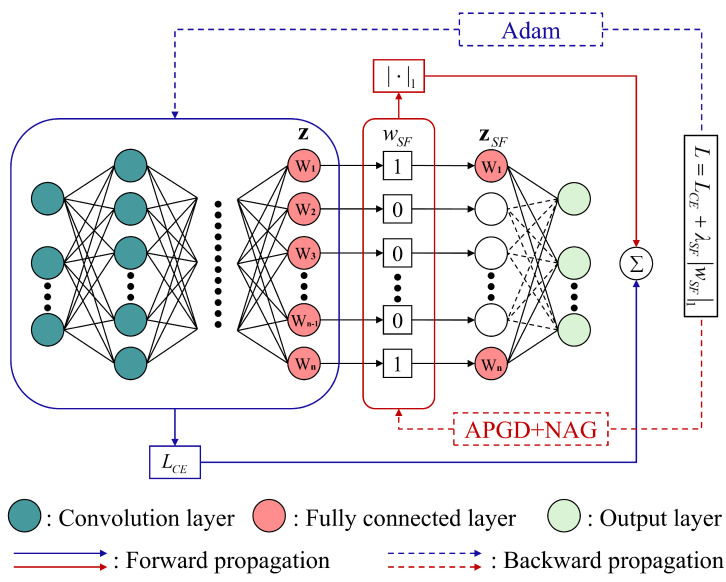
The framework of SFS (adapted from [30]).

The fundamental objective of the SFS framework is to impose regularized sparse constraints during the model training. Specifically, by incorporating the $\ell_{1}$-norm of the weights as a penalty term in the model’s loss function, it tends to produce a sparse weight matrix, thus achieving feature selection. It is important to note that this pruning operation is performed on a lightweight model. Therefore, conducting the pruning operation on convolution or batch normalization (BN) layers will result in an insufficient reduction of model parameters, significantly impairing accuracy. Inspired by the work in [30], our approach focuses on the FC layer. Specifically, as shown in Figure 5, we add the sparse parameter $w_{SF}$ to the original feature z, thereby obtaining the sparse feature $z_{SF}$, which can be expressed as

(12) $$z_{SF}=w_{SF}⊙z.$$

The sparse parameter $w_{SF}$ is initialized as $w_{SF}=\left\{1,1,\cdots ,1\right\}$, with the same dimensionality as the original feature z. During the model training process, due to the constraints of the regularized sparse loss function, the components of $w_{SF}$ corresponding to redundant features will be set to zero. Concurrently, the model’s parameters are reduced by pruning the weights associated with the redundant features.

To analyze the complexity of the sparse FC layer, a standard FC layer is introduced for comparison. Assuming the original feature z has an input neuron count of $N_{in}$, an output layer neuron count of $N_{out}$, and the number of redundant features set to zero in $w_{SF}$ is $N_{0}$. The complexity comparison between the standard and sparse FC layer is as follows:

(13) $$\begin{array}{cc} \frac{P_{spa}}{P_{std}} & =\frac{N_{out}\times \left(N_{in}-N_{0}\right)+N_{out}}{N_{out}\times N_{in}+N_{out}}=1-\frac{N_{0}}{N_{in}+1}, \end{array}$$

(14) $$\begin{array}{cc} \frac{T_{spa}}{T_{std}} & =\frac{N_{out}\times \left(N_{in}-N_{0}\right)+N_{out}\times \left(N_{in}-N_{0}-1\right)+N_{out}}{N_{out}\times N_{in}+N_{out}\times \left(N_{in}-1\right)+N_{out}}=1-\frac{N_{0}}{N_{in}}, \end{array}$$

where $P_{spa}/P_{std}$ and $T_{spa}/T_{std}$ represent the parameter and FLOPs ratios between sparse and standard FC layers, respectively. It can be deduced that, the greater the value of $N_{0}/N_{in}$, the lower the complexity of the sparsely connected layer.

### 3.3. APGD-NAG Optimization Algorithm and Training Procedure

Given a batch of training samples ${\left\{x_{i},y_{i}\right\}}_{i=1}^{B}$, where *B* denotes the batch size, the target loss function can be represented as

(15) $$\begin{array}{cc} L & =L_{CE}+R\left(w_{SF}\right)=-\frac{1}{B}\sum_{i}\log p_{y_{i}}+\lambda_{SF}{\left|w_{SF}\right|}_{1}, \end{array}$$

where $L_{CE}$ is the cross-entropy loss function, which effectively measures the performance of multi-classification models. $p_{y_{i}}$ represents the probability that the model predicts the correct category for the *i*-th sample. $R\left(w_{SF}\right)$ is the regularization term of the target loss function, also known as the penalty term. The model is encouraged to select a sparse solution by applying the $\ell_{1}$-norm to the sparse parameter $w_{SF}$. $\lambda_{SF}$ is the sparse factor, which balances the classification accuracy and sparsity of the model by controlling the strength of this coefficient. Therefore, choosing an appropriate $\lambda_{SF}$ is crucial.

The training process consists of forward propagation and back-propagation. As previously stated, the SFS framework is part of the forward propagation. This process is responsible for transforming input data into output results. In back-propagation, the neural network parameters w and the sparse parameter $w_{SF}$ are updated by using the target loss function L. Through iterations, the loss between the output results and the true labels is continuously reduced until the model’s classification accuracy and sparsity reach the desired level. Moreover, the neural network parameters w are optimized using the Adam algorithm, which can be expressed as [28]

(16) $$w^{k}=w^{k-1}-\eta \cdot \frac{{\hat{m}}_{k-1}}{\sqrt{{\hat{v}}_{k-1}}+\epsilon },\;k=1,2,\cdots ,K.$$

Here, η represents the learning rate and *k* is the number of training iterations, ${\hat{m}}_{k}$ and ${\hat{v}}_{k}$ are the bias-corrected first and second moment estimates of the gradients, respectively. ϵ is a small constant introduced to prevent division by zero in the denominator. However, the parameter ${\left|w_{SF}\right|}_{1}$ within the loss function L is a non-differentiable convex function, making the sparse parameter $w_{SF}$ unsuitable for direct optimization via the Adam algorithm. In cases where the objective loss function includes an $\ell_{1}$-norm penalty term, typically constitutes a least absolute shrinkage and selection operator (Lasso) problem, which is commonly addressed using the proximal gradient descent (PGD) algorithm. The fundamental idea behind this approach is to transform the minimization problem into its second-order approximation through a quadratic Taylor expansion. Formally, the optimization of $w_{SF}$ can be formulated as

(17) $$w_{SF}^{*}=\underset{z}{\arg\min}\left\{L_{CE}+R\left(w_{SF}\right)\right\},$$

(18) $$w_{SF}^{*}\approx \arg\min_{\theta }\left\{L_{CE}\left(w_{SF}\right)+\langle \nabla L_{CE}\left(w_{SF}\right),\left(\theta -w_{SF}\right)\rangle +\frac{\nabla^{2}L_{CE}\left(w_{SF}\right)}{2}\cdot {∥\theta -w_{SF}∥}_{2}^{2}+R\left(\theta \right)\right\}.$$

In (18), $L_{CE}\left(w_{SF}\right)$ is a constant term and can be omitted as it does not affect the optimization. $\nabla^{2}L_{CE}\left(w_{SF}\right)$ is the Hessian matrix of $L_{CE}$ at $w_{SF}$. Solving for this matrix would incur a high computational cost. According to the continuous Lipschitz condition [42], $dom\left(L_{CE}\right)=R^{n},\forall \;w_{SF}^{k},\;w_{SF}^{k-1}\in R^{n},\exists \;L>0$, satisfying

(19) $${∥\nabla L_{CE}\left(w_{SF}^{k}\right)-\nabla L_{CE}\left(w_{SF}^{k-1}\right)∥}_{2}\leq L{∥w_{SF}^{k}-w_{SF}^{k-1}∥}_{2}.$$

Therefore, the Lipschitz constant *L* can be regarded as an upper bound of $\nabla^{2}L_{CE}\left(w_{SF}\right)$ to simplify the solution process. Taking L=1/η, where η is the learning rate, also known as the iteration step size. The authors of [43] have proven that, when the iteration step size is set to the reciprocal of *L*, the loss converges can reach the fastest rate. Thus, (18) can be simplified to

(20) $$w_{SF}^{*}\approx \underset{\theta }{\arg\min}\left\{\frac{1}{2\eta }\cdot ∥\theta -{\left.\left[w_{SF}-\eta \nabla L_{CE}\left(w_{SF}\right)\right]\right|}_{2}^{2}+R\left(\theta \right)\right\}.$$

The expression in (20) can be reformulated using the proximal operator prox(·), thereby obtaining an explicit solution for $w_{SF}^{k}$. The proximal mapping is defined as follows:

(21) $${\mathrm{prox}}_{R,\eta }\left(w_{SF}\right)=\underset{\theta }{\mathrm{argmin}}\frac{1}{2\eta }{∥w_{SF}-\theta ∥}_{2}^{2}+R\left(\theta \right),$$

prox(·) is only related to R(θ), which can be solved through the iterative soft-thresholding algorithm (ISTA). Specifically, the soft-thresholding operator $S_{\eta \lambda_{SF}}\left(\cdot \right)$ acts as the gradient of the objective function containing the $\ell_{1}$-norm. The gradient update is projected onto the surface of the convex set, and gradient descent is employed to obtain the optimal solution. For better illustration, we shorten $w_{SF}^{k-1}-\eta \nabla L_{CE}\left(w_{SF}^{k-1}\right)$ as $h^{k-1}$, and reformulate the iterative optimization of $w_{SF}$ as

(22) $$\begin{array}{cc} w_{SF}^{k} & ={\mathrm{prox}}_{R,\eta }\left[w_{SF}^{k-1}-\eta \nabla L_{CE}\left(w_{SF}^{k-1}\right)\right]\\ \; & =S_{\eta \lambda_{SF}}\left[w_{SF}^{k-1}-\eta \nabla L_{CE}\left(w_{SF}^{k-1}\right)\right]\\ \; & =\mathrm{sgn}\left(h^{k-1}\right)\max\left(\left|h^{k-1}\right|-\eta \lambda_{SF},0\right). \end{array}$$

Subsequently, given the initialization $w_{SF}^{0}$, the approximate optimal solution $w_{SF}^{*}$ can be obtained through iterative refinement of *k*.

The APGD algorithm is a smarter improvement method for the PGD algorithm, with the key difference in the selection of the starting point for each iteration. Specifically, the APGD algorithm uses the results of the previous two iteration processes $w_{SF}^{k-1}$ and $w_{SF}^{k-2}$ to generate the starting point of the next iteration $w_{SF}^{k}$. This approach allows for faster convergence of the iterative process, which can be expressed as

(23) $$\left\{\begin{array}{c} w_{SF}^{k}=S_{\eta \lambda_{SF}}\left[d_{SF}^{k-1}-\eta \nabla L_{CE}\left(d_{SF}^{k-1}\right)\right]\\ d_{SF}^{k-1}=w_{SF}^{k-1}+\frac{k-2}{k+1}\left(w_{SF}^{k-1}-w_{SF}^{k-2}\right)\\ k=1,2,3,\ldots ,K \end{array}\right.$$

However, this formulation is not suitable for deep learning. This is because, besides the pass for updating $w_{SF}^{k}$, obtaining $\nabla L_{CE}\left(d_{SF}^{k-1}\right)$ requires an extra forward–backward computation, which is computationally expensive for deep neural networks. Thus, following [44,45], we reformulate APGD as the NAG-based method. Specifically, we define μ=(k−2)/(k+1); in practice, a very common value for μ is 0.9. And define $v^{k-1}=w_{SF}^{k-1}-w_{SF}^{k-2}$. Furthermore, we simplified the update of $w_{SF}^{k}$ by replacing $w_{SF}^{k-1}$ as $w_{SF}^{\prime k-1}=w_{SF}^{k-1}+\mu v^{k-1}$, following the modification of NAG in [46], which means employing the momentum at future points to replace that at the current point. The new parameters $w_{SF}^{\prime k-1}$ updates become

(24) $$\left\{\begin{array}{c} J^{k}=w_{SF}^{\prime k-1}-\eta \nabla L_{CE}\left(w_{SF}^{\prime k-1}\right)\\ v^{k}=S_{\eta \lambda_{SF}}\left(J^{k}\right)-w_{SF}^{\prime k-1}+\mu \cdot v^{k-1}\\ w_{SF}^{\prime k-1}=S_{\eta \lambda_{SF}}\left(J^{k}\right)+\mu \cdot v^{k-1}\\ k=1,2,3,\ldots ,K. \end{array}\right.$$

Due to the incorporation of the cumulative momentum $v^{k}$, there is no longer a need for a second forward–backward pass on $\nabla L_{CE}\left(w_{SF}^{\prime k-1}\right)$, thereby reducing the computational load of the model. The overall training process of the RC-SEI method is described in Algorithm 1.
**Algorithm 1:** Training Procedure of the RC-SEI method.**Require:***K*: Number of training iterations;*N*: Number of training samples;*C*: Number of batches;*B*: Number of Batchsize;$w,w_{SF}$: Parameters of neural network and sparse parameters;$z,z_{SF}$: Original features and sparse features;η: Learning rate;μ: Coefficient of momentum;$\lambda_{SF}$: Sparse factor;$\mathrm{Training}\mathrm{on}{\left\{x_{i},y_{i}\right\}}_{i=1}^{N}$**for** k=1 to *K* **do**   **for** c=1 to *C* **do**     **[Forward propagation]:**     Sampling a batch of training samples ${\left\{x_{i},y_{i}\right\}}_{i=1}^{B}$     Initialize $w_{SF}=\left\{1,1,\ldots ,1\right\}$     Extracting the Original features: z     Pruning the Original features: $z_{SF}=w_{SF}⊙z$     Obtaining the artificial labels:     **for** b=1 to *B* **do**        $\hat{y}=\arg\max_{y\in Y}f\left(y_{i}|x_{i};z_{SF}\right)$     **end for**     Calculating the loss: $L=L_{CE}+R\left(w_{SF}\right)$     **[Backward propagation]:**     Updating w by Adam algorithm:     $\begin{array}{c} w^{k}=w^{k-1}-\eta \cdot \frac{{\hat{m}}_{k-1}}{\sqrt{{\hat{v}}_{k-1}}+\epsilon } \end{array}$     Updating $w_{SF}$ by APGD-NAG algorithm:     $\begin{array}{c} \left\{\begin{array}{c} J^{k}=w_{SF}^{\prime k-1}-\eta \nabla L_{CE}\left(w_{SF}^{\prime k-1}\right)\\ v^{k}=S_{\eta \lambda_{SF}}\left(J^{k}\right)-w_{SF}^{\prime k-1}+\mu \cdot v^{k-1}\\ w_{SF}^{\prime k-1}=S_{\eta \lambda_{SF}}\left(J^{k}\right)+\mu \cdot v^{k-1}\\ k=1,2,3,\ldots ,K \end{array}\right. \end{array}$   **end for****end for**

## 4. Experimental Setup and Results

### 4.1. Experimental Methodology

In order to rigorously evaluate the proposed RS-SEI method, a series of experiments were conducted with a focus on its three core attributes: efficient design, network compression, and rapid convergence. The experimental setup is described first, followed by the evaluation of LCNet, SFS framework, and APGD-NAG.

#### 4.1.1. Datasets

For performance evaluation, two datasets were employed: a large-scale, real-world ADS-B radio signal dataset [32], and a Wi-Fi dataset [33]. The ADS-B dataset, collected using a USRP-SM200B with a center frequency of 1090 MHz, was gathered over one month in an open and unobstructed environment to minimize interference from surrounding buildings and other potential sources of radio noise. The Wi-Fi dataset, collected using a USRP-B210 with a center frequency of 2450 MHz, was acquired under both static channel conditions within the laboratory and dynamic channel conditions in the recreation area. ADS-B signals, typically encoded using pulse position modulation (PPM), are transmitted as 10-byte messages containing information such as aircraft identification, position, altitude, and velocity [47]. To ensure a rigorous evaluation and to focus on signal characteristics rather than aircraft-specific identifiers, the ICAO code (aircraft identification) was omitted from the ADS-B signals. The Wi-Fi dataset comprises frames conforming to the IEEE 802.11a standard [48], a prominent communication protocol within the IoT domain and a widely adopted wireless standard. Detailed characteristics of these datasets are summarized in Table 2.

#### 4.1.2. Baseline Models

For comparative analysis, we selected several representative signal recognition techniques, including two high-performance architectures (CVNN [24] and MCLDNN [35]) and two lightweight network designs (MCNet [34] and ULCNN [22]).

Specifically, CVNN is a nine-layer network employing CV convolution, which can directly process CV information and demonstrate excellent performance in signal classification. MCLDNN uses a multi-channel learning framework combined with long short-term Memory (LSTM) units to extract temporal features, enabling efficient modulated signal recognition. MCNet reduces convolution layer parameters through asymmetric convolution kernels and M-blocks, further enhancing classification accuracy with skip connections between multiple M-blocks. ULCNN employs depth-wise separable convolution and cross-layer feature fusion, significantly reducing parameters in convolution and FC layers, sharing conceptual similarities with our proposed method. To ensure a fair comparison, we applied the SFS framework to compress all networks while preserving their core functionalities. Detailed simulation parameters are listed in Table 3.

### 4.2. LCNet Evaluation

#### 4.2.1. LCNet vs. StdNet

To better evaluate the performance of LCNet, we established StdCNet as a baseline. Structurally, StdCNet is identical to LCNet, except it lacks the lightweight design elements. The architecture of StdCNet comprises two primary modules: the Std_IQCF module using RV convolution, and the Std_LCM composed of standard convolution. The detailed architecture of StdCNet is presented in Table 4.

An evaluation was conducted to assess the performance of LCNet and StdCNet on the ADS-B and Wi-Fi datasets. The experimental results, detailed in Table 5, demonstrate the superior performance of LCNet in terms of both accuracy and complexity. Specifically, on the ADS-B dataset, LCNet achieved a 4.3% accuracy gain over StdCNet, while simultaneously reducing parameters by 71.57% and FLOPs by 74.82%. Similarly, on the Wi-Fi dataset, LCNet exhibited a 0.95% accuracy improvement compared to StdCNet, coupled with a 65.96% reduction in parameters and a 74.81% reduction in FLOPs. These results highlight that a well-designed, efficient architecture can maintain high model accuracy while substantially reducing model complexity.

#### 4.2.2. Ablation Study

To investigate the impact of varying LCM layer depth and incorporating the DSECA technique within the LCNet architecture on model performance, five comparative experiments were conducted on the ADS-B and Wi-Fi datasets. The primary evaluation metrics included accuracy, parameter, and FLOPs. Additionally, the number of neurons $N_{in}$ in the FC layer (z in Figure 5) was recorded, which aids in interpreting the experimental results.

As illustrated in Table 6, the model’s FLOPs increase with the number of LCM layers, whereas the number of parameters initially decreases and then increases. This is attributed to the fact that the output of the final LCM layer serves as the input to the FC layer. Consequently, adding more LCM layers enhances the depth of the convolution layers while simultaneously reducing the number of neurons in the FC layer. These competing effects ultimately influence the overall model complexity.

In addition, the model’s classification performance is contingent on the characteristics of the dataset. Specifically, when evaluated on the ADS-B dataset, classification accuracy improves as the number of LCM layers increases, reaching a maximum of 98.2% at nine layers. Conversely, classification performance on the Wi-Fi dataset remains consistently high, irrespective of the number of LCM layers. This discrepancy can be attributed to the Wi-Fi dataset’s superior sample length and quantity relative to the ADS-B dataset, which facilitates enhanced model performance.

Furthermore, the inclusion of the DSECA module substantially enhances the model’s performance on the ADS-B dataset, particularly when the number of LCM layers is relatively small. For instance, at six LCM layers, utilizing DSECA results in a performance gain of 11%. However, on the Wi-Fi dataset, the performance improvement from DSECA is marginal, and may even cause a slight degradation. Nevertheless, given the minimal number of parameters in DSECA (only eight), its cost-effectiveness is considerable.

In conclusion, based on the ablation study results of LCNet across both datasets, and considering that the subsequent SFS framework will apply significant compression to the FC layer, the architecture employing seven LCM layers in conjunction with DSECA is considered the optimal choice.

### 4.3. SFS Framework Evalution

#### 4.3.1. Sparse Factor Impact

In this section, an exploration was conducted into the impact of sparse factors $\lambda_{SF}$ on model performance, where $\lambda_{SF}=0$ represents no compression applied to the FC layer. Before delving into these results, it is necessary to introduce three key metrics for evaluating the compression efficiency of the SFS framework: (1) Feature sparsity of the FC layer ($R_{z}$); (2) Compression ratio of the parameters in the FC layer ($R_{P}$); (3) Reduction ratio of FLOPs in the FC layer ($R_{T}$). These metrics are defined as follows:

(25) $$R_{z}=\frac{N_{in}-N_{out}}{N_{in}}\times 100\%,$$

(26) $$R_{P}=\frac{Param_{original}-Param_{slim}}{Param_{original}}\times 100\%,$$

(27) $$R_{T}=\frac{FLOPs_{original}-FLOPs_{slim}}{FLOPs_{original}}\times 100\%.$$

Table 7 shows that, as $\lambda_{SF}$ increases, all three key metrics correspondingly increase, with the compression ratio reaching up to 99%. However, such extensive compression often leads to a notable decline in accuracy. Specifically, on the ADS-B dataset, accuracy decreases significantly as $\lambda_{SF}$ increases, resulting in a 12.60% reduction when $\lambda_{SF}=15$. In contrast, on the Wi-Fi dataset, accuracy remains relatively stable with the increase in $\lambda_{SF}$; in fact, there is an increase of 0.10% in accuracy when $\lambda_{SF}=1$.

This phenomenon can be attributed to two primary factors: First, whereas a high $\lambda_{SF}$ promotes convergence of the objective function towards model sparsity, the superior quality of the Wi-Fi dataset enhances model robustness. Second, the SFS framework eliminates redundant features, allowing the model to focus on important features, which contributes to the increase in accuracy post-compression.

Overall, selecting an appropriate $\lambda_{SF}$ can significantly reduce the complexity of the FC layer while preserving high accuracy. Furthermore, to mitigate the accuracy loss after compression, we introduce retraining to restore the performance of the sparse model.

#### 4.3.2. Retraining Efficacy

In the previous section, experimental findings indicated that augmenting the parameter $\lambda_{SF}$ enhances feature sparsity. However, this phenomenon concomitantly negatively impacts the model’s classification performance. To address this accuracy loss, a retraining strategy was employed. During the retraining phase, we removed the sparse regularization and utilized the initial training parameters for transfer learning.

As demonstrated in Table 7, the sparse model’s precision underwent a substantial enhancement following retraining. Specifically, on the ADS-B dataset, the retraining accuracy reached 99.40% when $\lambda_{SF}=12$, which is higher than the original 99.30%. On the Wi-Fi dataset, the model returned to its original accuracy of 99.90% when $\lambda_{SF}=5,10,12,15$. Given the performance and complexity of the LCNet under different sparsity factors, we selected $\lambda_{SF}=12$ and $\lambda_{SF}=10$ as the optimal sparsity factors for the ADS-B and Wi-Fi datasets, respectively.

**Figure 6:**
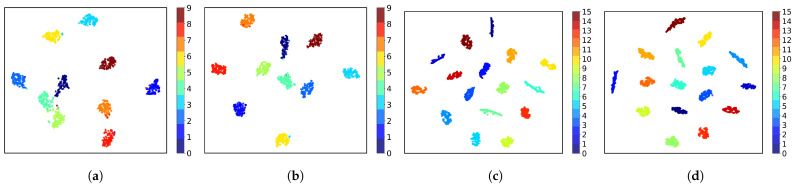
t-SNE visualization of LCNet feature distribution before and after retraining. (**a**) Before retraining on ADS-B, (**b**) after retraining on ADS-B, (**c**) before retraining on Wi-Fi, (**d**) after retraining on Wi-Fi.

We used t-SNE [49] to visualize the feature distribution before and after retraining for both datasets. In addition, the silhouette coefficient was introduced as a quantitative metric to evaluate the performance of feature clustering. This metric ranges from [−1,1], with values closer to 1 indicating better clustering. The feature visualizations are shown in Figure 6, and the silhouette coefficients for (a), (b), (c), and (d) were calculated as 0.5075, 0.5328, 0.6904, and 0.7137, respectively. The results strongly demonstrate that retraining improves the intercategory dispersion and intracategory compactness.

#### 4.3.3. Comparative Network Analysis

In order to further investigate the effectiveness and limitations of the SFS framework, its application was expanded to multiple networks, and experiments were conducted. A thorough analysis of the data presented in Table 8 yielded the following three primary conclusions:

1. Effectiveness: The SFS framework can be readily applied to both high-precision and lightweight models, with the compressed models largely maintaining comparable accuracy levels to their uncompressed counterparts. Specifically, in accuracy tests on two datasets, MCNet experienced only a 0.90% decrease in accuracy on the ADS-B dataset, whereas CVNN saw decreases of 0.20% and 0.45% on the ADS-B and Wi-Fi datasets, respectively. The accuracy of other models either remained consistent or exhibited an improvement in post-compression performance. Notably, our proposed LCNet achieved the advanced level of accuracy on both datasets, with performance levels of 99.40% and 99.90%, respectively.
2. Limitations: The effectiveness of model compression methods based on the SFS framework is contingent on the model’s complexity. For highly complex models, the parameter compression rate tends to be lower. However, the employment of efficiently designed ULCNN and our proposed LCNet can yield superior parameter compression results.
3. Extension: It is noteworthy that SFS can compress over 99% of the parameters and FLOPs in the FC layer. Consequently, the parameter compression rates and FLOPs reductions shown in Table 8 accurately reflect the proportion of complexity within the FC layer relative to the overall model complexity. This indicates that there is a significant amount of redundancy in network models beyond the FC layer. To minimize overall model redundancy, building upon the RC-SEI method proposed in this paper, we suggest designing lightweight network models following these steps:
  - Initially, a high-performance neural network model should be trained without regard for complexity.
  - Subsequently, utilizing the dark knowledge from the initial step and efficient network design principles, redesign a compact neural network model.
  - Finally, applying the SFS framework to massively compress the complexity of the FC layer.

#### 4.3.4. Complexity Analysis

In this paper, two metrics are presented for the evaluation of model complexity. The number of parameters is used to describe the model’s spatial complexity, while FLOPs is used to describe its computational complexity. As shown in Table 8, our LCNet method has fewer than 34,000 parameters, which is approximately 1/20 of that of MCLDNN, and the least among all the methods. Furthermore, the FLOPs of our method are also significantly lower than those of other methods.

Considering that FLOPs alone cannot directly reflect the model’s inference speed, we further measured the pre-sample inference time for different batch sizes on a GPU as a supplementary metric. The test results presented Table 9 demonstrate that our method exhibits the fastest inference speed on both the ADS-B and Wi-Fi datasets. The inference speed rankings of the other models are generally consistent with the FLOPs metric, with the exception of MCNet. While MCNet has a higher FLOPs count, its highly parallel model architecture allows it to achieve an inference speed performance second only to our method.

### 4.4. APGD-NAG Evaluation

**Figure 7:**
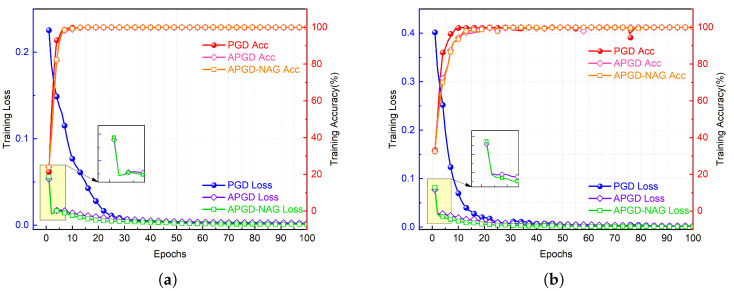
Training accuracy and loss comparison of PGD, APGD, and APGD-NAG. (**a**,**b**) correspond to ADS-B and Wi-Fi datasets, respectively.

PGD, APGD, and APGD-NAG can be regarded as variations of gradient descent algorithm, momentum algorithm, and NAG algorithm, respectively. Among these, APGD-NAG enhances the precision and efficiency of gradient updates by incorporating a forward-looking gradient estimation. This is validated in Figure 7, where the convergence rate of APGD and APGD-NAG is significantly faster than that of the PGD algorithm. Furthermore, the final loss after optimization with APGD-NAG is lower than that of APGD, indicating that APGD-NAG performs better in model optimization.

**Figure 8:**
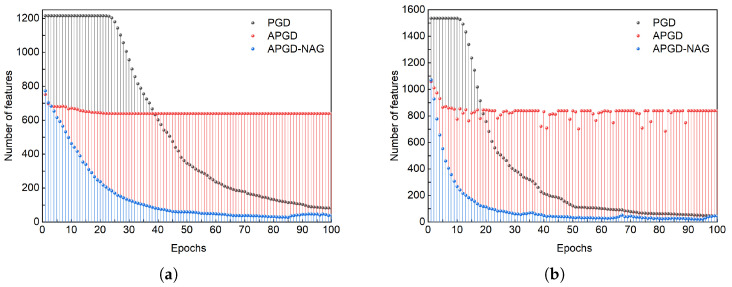
Trend of sparse feature count during training with PGD, APGD, and APGD-NAG. (**a**,**b**) correspond to ADS-B and Wi-Fi datasets, respectively.

To evaluate the impact of these three algorithms on model sparsity, we analyzed the trend of sparse feature changes during their iterative processes under the same $\lambda_{SF}$. As illustrated in Figure 8, both PGD and APGD-NAG effectively converge the model towards the most sparse direction, with APGD-NAG converging more rapidly. Additionally, APGD exhibited a promising convergence speed during the initial epochs, but subsequently fell into a local optimum, causing the model to cease converging towards the sparse direction. In summary, APGD-NAG not only accelerates the convergence of losses but also encourages the model to select a sparse solution.

## 5. Conclusions

In this paper, we propose a novel RC-SEI method that integrates efficient design and model compression strategies. This approach not only exhibits low FLOPs and a reduced parameter count but also accelerates training and enhances feature sparsity. Furthermore, we conducted extensive experiments using ADS-B and Wi-Fi datasets. The experimental results demonstrate that our proposed LCNet achieves superior recognition performance while maintaining the smallest parameter scale, compared to other state-of-the-art models in the same category. Based on the effectiveness and limitations of the SFS framework, we have also formulated a set of standardized procedures for constructing lightweight network models, aiming to minimize model complexity and redundancy. Future work will focus on improving the robustness of radiation source signal identification algorithms against noise and channel effects [50], and exploring incremental learning [51] to quickly identify new and emerging signal categories.

## Figures and Tables

**Table 1:** The structure of LCNet.

Module	Structure	Layers
IQCF	CVConv1D + CVReLU + CVBN	1
LCM	Separable Conv1D + ReLU + BN + Channel shuffle + DSECA	7
Classifier	Flatten + ReLU + SoftMax	1

**Table 2:** Details of the datasets.

Items	ADS-B	Wi-Fi
Format	IQ	IQ
Number of categories	10	16
Sample length	4800	6000
Number of samples	4080	10,000
Signal transmitter	ADS-B-OUT	X310-USRP-SDR
Signal receiver	USRP-SM200B	USRP-B210
Carrier frequency	1090 MHz	2450 MHz

**Table 3:** Simulation parameters.

Items	ADS-B	Wi-Fi
Training samples	2772	7200
Validation samples	308	800
Test samples	1000	2000
Sparse factor	{0, 1, 2, 5, 10, 12, 15}	
Epochs	100	
Batch size	256	
Learning rate of Adam	0.01	
Learning rate of APGD-NAG	0.001	
Platform	NVIDIA GeForce RTX 3090 GPU	
Environment	PyTorch V1.10.1, python 3.6.13	

**Table 4:** The structure of StdCNet.

Module	Structure	Number of Layers
Std_IQCF	Conv2D + ReLU + BN	×1
Std_LCM	Conv1D + ReLU + BN + DSECA	×7
Classifier	Flatten + ReLU + SoftMax	×1

**Table 5:** Comparison of accuracy, parameters, and FLOPs between LCNet and StdCNet.

Network (Dataset)	Accuracy	Parameters	FLOPs/M
StdCNet (ADS-B)	95.00%	157,445	50.95
LCNet (ADS-B)	99.30% (↑4.3%)	45,570 (↓71.57%)	12.83 (↓74.82%)
StdCNet (Wi-Fi)	98.95%	169,867	63.708
LCNet (Wi-Fi)	99.90% (↑0.95%)	57,992 (↓65.96%)	16.05 (↓74.81%)

**Table 6:** Ablation study results of the LCNet component LCM and DSECA.

Dataset	Layers of LCM	Numbers of $\;N_{\mathrm{in}}$	Without DSECA	With DSECA	Parameters	FLOPs/M
ADS-B	6	2432	87.40%	98.40% (↑11.00%)	53,050	12.75
	7	1216	93.00%	99.30% (↑6.30%)	45,570	12.83
	8	640	97.70%	99.30% (↑1.60%)	44,490	12.87
	9	320	98.20%	99.10% (↑0.90%)	45,970	12.89
	10	160	97.90%	98.60% (↑0.70%)	49,370	12.91
Wi-Fi	6	3072	99.85%	99.80% (↓0.05%)	76,864	15.60
	7	1536	99.80%	99.90% (↑1.00%)	57,992	16.05
	8	768	100.0%	99.95% (↓0.05%)	50,384	16.09
	9	384	98.90%	99.70% (↑0.80%)	48,920	16.12
	10	192	99.20%	99.70% (↑0.50%)	50,528	16.13

**Table 7:** Comprehensive evaluation of LCNet under different sparse factors.

Dataset	$\lambda_{\mathrm{SF}}$	$N_{0}$ ($R_{z}$)	Accuracy	Accuracy After Retraining	Parameters ($R_{P}$)	FLOPs ($R_{T}$)
ADS-B	0	1216	99.30%		12,170	24,320
	1	249 (↓79.52%)	98.00% (↓1.30%)	99.10% (↑1.10%)	2500 (↓79.46%)	4980 (↓79.52%)
	2	32 (↓97.37%)	97.80% (↓1.50%)	98.90% (↑1.10%)	330 (↓97.29%)	640 (↓97.37%)
	5	59 (↓95.15%)	98.60% (↓0.70%)	99.20% (↑0.60%)	600 (↓95.07%)	1180 (↓95.15%)
	10	12 (↓99.01%)	93.20% (↓6.10%)	98.50% (↑5.30%)	130 (↓98.93%)	240 (↓99.01%)
	12	10 (↓99.18%)	93.40% (↓5.90%)	99.40% (↑6.00%)	110 (↓99.10%)	200 (↓99.18%)
	15	15 (↓98.77%)	86.70% (↓12.6%)	99.10% (↑12.40%)	160 (↓98.69%)	300 (↓98.77%)
Wi-Fi	0	1536	99.90%		24,592	49,152
	1	74 (↓95.18%)	100.00% (↑0.10%)	99.85% (↓0.15%)	1200 (↓95.12%)	2368 (↓95.18%)
	2	46 (↓97.01%)	97.30% (↓2.60%)	99.80% (↑2.50%)	752 (↓96.94%)	1472 (↓97.01%)
	5	16 (↓98.96%)	95.95% (↓3.95%)	99.90% (↑3.95%)	272 (↓98.89%)	512 (↓98.96%)
	10	9 (↓99.41%)	99.20% (↓0.70%)	99.90% (↑0.70%)	160 (↓98.25%)	288 (↓99.41%)
	12	10 (↓99.35%)	99.35% (↓0.55%)	99.90% (↑0.55%)	176 (↓99.28%)	320 (↓99.35%)
	15	8 (↓99.48%)	98.60% (↓1.30%)	99.90% (↑1.30%)	144 (↓99.41%)	256 (↓99.48%)

**Table 8:** Comprehensive evaluation of LCNet and baseline models.

Dataset	Model	$\lambda_{\mathrm{SF}}$	Accuracy	Parameters	FLOPs/M
ADS-B	LCNet	0	99.30%	45,570	12.83
		12	99.40% (↑0.10%)	33,510 (↓26.46%)	12.82 (↓0.01)
	MCNet	0	99.30%	289,002	209.23
		2	98.40% (↓0.90%)	284,102 (↓1.70%)	209.22 (↓0.01)
	ULCNN	0	98.40%	56,906	25.70
		12	98.50% (↑0.10%)	50,681 (↓10.94%)	25.69 (↓0.01)
	CVNN	0	98.60%	407,562	242.01
		12	98.40%(↓0.20%)	398,722 (↓2.17%)	242.00 (↓0.01)
	MCLDNN	0	96.60%	655,758	376.85
		5	96.80% (↑0.20%)	650,798 (↓0.76%)	376.84 (↓0.01)
Wi-Fi	LCNet	0	99.90%	57,992	16.05
		15	99.90% (↑0.00%)	33,544 (↓42.16%)	16.03 (↓0.02)
	MCNet	0	99.5%0	292,080	261.46
		10	99.75% (↑0.25%)	284,048 (↓2.75%)	261.45 (↓0.01)
	ULCNN	0	98.95%	57,680	32.13
		2	99.95% (↑0.10%)	53,549 (↓7.16%)	32.12 (↓0.01)
	CVNN	0	99.95%	417,040	302.85
		15	99.50% (↓0.45%)	398,782 (↓4.38%)	302.83 (↓0.02)
	MCLDNN	0	98.80%	658,836	471.25
		12	99.50% (↑0.70%)	650,756 (↓1.23%)	471.24 (↓0.01)

**Table 9:** Inference time of per sample on GPU for different networks and batch sizes.

Network	Inference Time of Per Sample in Different Batch Sizes (ms)			
	1	10	100	1000
LCNet	8.221/7.753	0.839/0.905	0.087/0.085	0.044/0.055
MCNet	10.293/10.344	0.993/1.027	0.108/0.112	0.076/0.091
ULCNN	9.824/9.486	1.008/0.992	0.104/0.123	0.092/0.110
CVNN	12.872/11.129	1.182/1.182	0.167/0.202	0.173/0.222
MCLDNN	24.899/29.891	2.656/3.064	0.370/0.425	0.225/0.278

Note: Values left/right of the slash (/) represent results on ADS-B and Wi-Fi datasets, respectively.

## Data Availability

The datasets and code are available at: https://github.com/Mengtao-Wang/RC-SEI (accessed on 31 March 2025).

## References

[1] Rajora R., Rajora A., Sharma B., Aggarwal P., Thapliyal S. Sensing the Future: Challenges and Trends in IoT Sensor Technology. Proceedings of the 4th International Conference on Innovative Practices in Technology and Management, ICIPTM.

[2] Chakrabarty R., Karmakar R., Das N.K., Shivam S., Mondal I. The Future of Real-Time Remote Monitoring: The Role of Low-Cost IoT Sensor Systems. Proceedings of the 7th International Conference on Electronics, Materials Engineering & Nano-Technology (IEMENTech).

[3] Yin X., Wang S., Shahzad M., Hu J. (2021). An IoT-Oriented Privacy-Preserving Fingerprint Authentication System. IEEE Internet Things J..

[4] Sun P., Shen S., Wan Y., Wu Z., Fang Z., Gao X.-Z. (2024). A Survey of IoT Privacy Security: Architecture, Technology, Challenges, and Trends. IEEE Internet Things J..

[5] Meng R., Xu B., Xu X., Sun M., Wang B., Han S., Lv S., Zhang P. (2024). A Survey of Machine Learning-Based Physical-Layer Authentication in Wireless Communications. J. Netw. Comput. Appl..

[6] Tyler J.H., Fadul M.K.M., Reising D.R. (2023). Considerations, Advances, and Challenges Associated with the Use of Specific Emitter Identification in the Security of Internet of Things Deployments: A Survey. Information.

[7] Huan X., Hao Y., Miao K., He H., Hu H. (2023). Carrier Frequency Offset in Internet of Things Radio Frequency Fingerprint Identification: An Experimental Review. IEEE Internet Things J..

[8] Diwakaran S., Vijayakumari P., Kuppusamy P.G., Kosalendra E., Krishnamoorthi K. A Safe and Reliable Digital Fingerprint Recognition Method for Internet of Things (IoT) Devices. Proceedings of the International Conference on Artificial Intelligence and Knowledge Discovery in Concurrent Engineering (ICECONF).

[9] Farha F., Ning H., Ali K., Chen L., Nugent C. (2020). SRAM-PUF-Based Entities Authentication Scheme for Resource-Constrained IoT Devices. IEEE Internet Things J..

[10] Ahmed A., Quoitin B., Gros A., Moeyaert V. (2024). A Comprehensive Survey on Deep Learning-Based LoRa Radio Frequency Fingerprinting Identification. Sensors.

[11] Wan H., Wang Q., Fu X., Wang Y., Zhao H., Lin Y., Sari H., Gui G. (2024). VC-SEI: Robust Variable-Channel Specific Emitter Identification Method Using Semi-Supervised Domain Adaptation. IEEE Trans. Wirel. Commun..

[12] Soltanieh N., Norouzi Y., Yang Y., Karmakar N.C. (2020). A Review of Radio Frequency Fingerprinting Techniques. IEEE J. Radio Freq. Identif..

[13] Tu Y., Zhang Z., Li Y., Wang C., Xiao Y. (2019). Research on the Internet of Things Device Recognition Based on RF-Fingerprinting. IEEE Access.

[14] Chen S., Xie F., Chen Y., Song H., Wen H. Identification of Wireless Transceiver Devices Using Radio Frequency (RF) Fingerprinting Based on STFT Analysis to Enhance Authentication Security. Proceedings of the IEEE 5th International Symposium on Electromagnetic Compatibility (EMC-Beijing).

[15] Saadouni C., El Jaouhari S., Tamani N., Ziti S., Mroueh L., El Bouchti K. (2025). Identification Techniques in the Internet of Things: Survey, Taxonomy and Research Frontier. IEEE Commun. Surv. Tutor..

[16] Yu J., Hu A., Li G., Peng L. (2019). A Robust RF Fingerprinting Approach Using Multisampling Convolutional Neural Network. IEEE Internet Things J..

[17] Han G., Xu Z., Zhu H., Ge Y., Peng J. (2023). A Two-Stage Model Based on a Complex-Valued Separate Residual Network for Cross-Domain IIoT Devices Identification. IEEE Trans. Ind. Inf..

[18] Wang Y., Gui G., Lin Y., Wu H.-C., Yuen C., Adachi F. (2022). Few-Shot Specific Emitter Identification via Deep Metric Ensemble Learning. IEEE Internet Things J..

[19] Fu X., Peng Y., Liu Y., Lin Y., Gui G., Gacanin H., Adachi F. (2023). Semi-Supervised Specific Emitter Identification Method Using Metric-Adversarial Training. IEEE Internet Things J..

[20] Huang S., Guo L., Fu X., Peng Y., Guo Y., Wang Y., Zhang Q., Gui G., Sari H. (2024). Open-Set Specific Emitter Identification Leveraging Enhanced Metric Denoising Auto-Encoders. IEEE Internet Things J..

[21] Guo J., Wang J., Wen C.-K., Jin S., Li G.Y. (2020). Compression and Acceleration of Neural Networks for Communications. IEEE Wirel. Commun..

[22] Guo L., Wang Y., Liu Y., Lin Y., Zhao H., Gui G. (2024). Ultra Convolutional Neural Network for Automatic Modulation Classification in Internet of Unmanned Aerial Vehicles. IEEE Internet Things J..

[23] Chang S., Yang Z., He J., Li R., Huang S., Feng Z. (2023). A Fast Multi-Loss Learning Deep Neural Network for Automatic Modulation Classification. IEEE Trans. Cogn. Commun. Netw..

[24] Dong P., He C., Gao S., Zhou F., Wu Q. (2024). Edge-Learning-Based Collaborative Automatic Modulation Classification for Hierarchical Cognitive Radio Networks. IEEE Internet Things J..

[25] Jiang J., Huang H. (2024). Complex-Valued Soft-Log Threshold Reweighting for Sparsity of Complex-Valued Convolutional Neural Networks. Neural Net..

[26] Franco N.R., Brugiapaglia S. (2024). A Practical Existence Theorem for Reduced Order Models Based on Convolutional Autoencoders. arXiv.

[27] Zhang Y., Peng Y., Sun J., Gui G., Lin Y., Mao S. (2023). GPU-Free Specific Emitter Identification Using Signal Feature Embedded Broad Learning. IEEE Internet Things J..

[28] Dong B., Liu Y., Gui G., Fu X., Dong H., Adebisi B., Gacanin H., Sari H. (2022). A Lightweight Decentralized-Learning-Based Automatic Modulation Classification Method for Resource-Constrained Edge Devices. IEEE Internet Things J..

[29] Hua M., Zhang Y., Sun J., Adebisi B., Ohtsuki T., Gui G., Wu H.-C., Sari H. (2023). Specific Emitter Identification Using Adaptive Signal Feature Embedded Knowledge Graph. IEEE Internet Things J..

[30] Tao M., Fu X., Lin Y., Wang Y., Yao Z., Shi S., Gui G. Resource-Constrained Specific Emitter Identification Using End-to-End Sparse Feature Selection. Proceedings of the IEEE Global Communications Conference (GLOBECOM).

[31] Li S., Chen J., Liu S., Zhu C., Tian G., Liu Y. (2025). MCMC: Multi-Constrained Model Compression via One-Stage Envelope Reinforcement Learning. IEEE Trans. Neural Netw. Learn. Syst..

[32] Ya T., Yun L., Haoran Z., Ju Z., Yu W., Guan G., Shiwen M. (2022). Large-Scale Real-World Radio Signal Recognition with Deep Learning. Chin. J. Aeronaut..

[33] Sankhe K., Belgiovine M., Zhou F., Riyaz S., Ioannidis S., Chowdhury K. ORACLE: Optimized Radio Classification Through Convolutional Neural Networks. Proceedings of the IEEE Conference on Computer Communications (INFOCOM).

[34] Huynh-The T., Hua C.-H., Pham Q.-V., Kim D.-S. (2020). MCNet: An Efficient CNN Architecture for Robust Automatic Modulation Classification. IEEE Commun. Lett..

[35] Xu J., Luo C., Parr G., Luo Y. (2020). A Spatiotemporal Multi-Channel Learning Framework for Automatic Modulation Recognition. IEEE Wirel. Commun. Lett..

[36] Wang M., Fang S., Fan Y., Li J., Zhao Y., Wang Y. (2024). An Ultra Lightweight Neural Network for Automatic Modulation Classification in Drone Communications. Sci. Rep..

[37] Tu Y., Lin Y., Hou C., Mao S. (2020). Complex-Valued Networks for Automatic Modulation Classification. IEEE Trans. Veh. Technol..

[38] Xiao C., Yang S., Feng Z. (2023). Complex-Valued Depth-Wise Separable Convolutional Neural Network for Automatic Modulation Classification. IEEE Trans. Instrum. Meas..

[39] Sandler M., Howard A., Zhu M., Zhmoginov A., Chen L.-C. MobileNetV2: Inverted Residuals and Linear Bottlenecks. Proceedings of the IEEE Conference on Computer Vision and Pattern Recognition (CVPR).

[40] Wang Q., Wu B., Zhu P., Li P., Zuo W., Hu Q. ECA-Net: Efficient Channel Attention for Deep Convolutional Neural Networks. Proceedings of the IEEE/CVF Conference on Computer Vision and Pattern Recognition (CVPR).

[41] Lu X., Tao M., Fu X., Gui G., Ohtsuki T., Sari H. Lightweight Network Design Based on ResNet Structure for Modulation Recognition. Proceedings of the IEEE Vehicular Technology Conference (VTC).

[42] Bubeck S. (2015). Convex Optimization: Algorithms and Complexity. Found. Trends Mach. Learn..

[43] Beck A., Teboulle M. (2009). A Fast Iterative Shrinkage-Thresholding Algorithm for Linear Inverse Problems. SIAM J. Imaging Sci..

[44] Huang Z., Wang N. Data-Driven Sparse Structure Selection for Deep Neural Networks. Proceedings of the European Conference on Computer Vision (ECCV).

[45] Yang Z., Bao W., Yuan D., Tran N.H., Zomaya A.Y. (2022). Federated Learning with Nesterov Accelerated Gradient. IEEE Trans. Parallel Distrib. Syst..

[46] Bengio Y., Boulanger-Lewandowski N., Pascanu R. Advances in Optimizing Recurrent Networks. Proceedings of the IEEE International Conference on Acoustics, Speech, and Signal Processing (ICASSP).

[47] Pearce N., Duncan K.J., Jonas B. Signal Discrimination and Exploitation of ADS-B Transmission. Proceedings of the SoutheastCon.

[48] IEEE (1999). IEEE Standard for Information Technology—Telecommunications and Information Exchange Between Systems—Local and Metropolitan Area Networks—Specific Requirements Part 11: Wireless LAN Medium Access Control (MAC) and Physical Layer (PHY) Specifications Amendment 1: High-Speed Physical Layer in the 5 GHz Band.

[49] Cai T.T., Ma R. (2022). Theoretical Foundations of T-SNE for Visualizing High-Dimensional Clustered Data. J. Mach. Learn. Res..

[50] Han Y., Chen X., Wang M., Shi L., Feng Z. (2024). GP-DGECN: Geometric Prior Dynamic Group Equivariant Convolutional Networks for Specific Emitter Identification. IEEE Open J. Commun. Soc..

[51] Li D., Qi J., Hong S., Deng P., Sun H. (2023). A Class-Incremental Approach with Self-Training and Prototype Augmentation for Specific Emitter Identification. IEEE Trans. Inf. Forensics Secur..

